# Butyrate augments interferon-**α**-induced S phase accumulation and persistent tyrosine phosphorylation of cdc2 in K562 cells

**DOI:** 10.1038/sj.bjc.6690163

**Published:** 1999-03

**Authors:** T Miyachi, M Adachi, Y Hinoda, K Imai

**Affiliations:** The First Department of Internal Medicine, Sapporo Medical University School of Medicine, Chuo-ku, Sapporo 060-8543, Japan

**Keywords:** butyrate, IFN-α, S phase accumulation, cdc2, tyrosine phosphorylation

## Abstract

Interferon-α (IFN-α) is a clinically useful cytokine for treatment of a variety of cancers, including chronic myelocytic leukaemia (CML). Most CML cells are sensitive to IFN-α; however, its biological effects on leukaemic cells are incompletely characterized. Here, we provide evidence that IFN-α induces a significant increase in the S phase population in human CML leukaemic cell line, K562, and that the S phase accumulation was augmented by sodium butyrate. In contrast, neither sodium butyrate alone, nor sodium butyrate plus IFN-γ, affected the cell cycle in K562 cells. These data suggest that the effect of sodium butyrate depended upon IFN-α-mediated signalling. The ability of leukaemic cells to exhibit the S phase accumulation after stimulation by IFN-α plus sodium butyrate correlated well with persistent tyrosine phosphorylation of cdc2, whereas treatment with IFN-γ plus sodium butyrate did not affect its phosphorylation levels. Considering that dephosphorylation of cdc2 leads to entry to the M phase, the persistent tyrosine phosphorylation of cdc2 may be associated with the S phase accumulation induced by IFN-α and sodium butyrate. In addition, another human CML leukaemic cell line, MEG-01, also showed the S phase accumulation after stimulation with IFN-α plus sodium butyrate. Taken together, our studies reveal a novel effect of sodium butyrate on the S phase accumulation and suggest its clinical application for a combination therapy with IFN-α, leading to a great improvement of clinical effects of IFN-α against CML cells. © 1999 Cancer Research Campaign


*British Journal of Cancer* (1999) **79**(7/8), 1018–1024
© 1999 Cancer Research Campaign
Article no. bjoc.1998.0163

### Butyrate augments interferon-α-induced S phase accumulation and persistent tyrosine phosphorylation of cdc2 in K562 cells

**T Miyachi, M Adachi, Y Hinoda and K Imai**

The First Department of Internal Medicine, Sapporo Medical University School of Medicine, S1 W16, Chuo-ku, Sapporo 060-8543, Japan

**Summary** Interferon-α (IFN-α) is a clinically useful cytokine for treatment of a variety of cancers, including chronic myelocytic leukaemia (CML). Most CML cells are sensitive to IFN-α; however, its biological effects on leukaemic cells are incompletely characterized. Here, we provide evidence that IFN-α induces a significant increase in the S phase population in human CML leukaemic cell line, K562, and that the S phase accumulation was augmented by sodium butyrate. In contrast, neither sodium butyrate alone, nor sodium butyrate plus IFN-γ, affected the cell cycle in K562 cells. These data suggest that the effect of sodium butyrate depended upon IFN-α-mediated signalling. The ability of leukaemic cells to exhibit the S phase accumulation after stimulation by IFN-α plus sodium butyrate correlated well with persistent tyrosine phosphorylation of cdc2, whereas treatment with IFN-γ plus sodium butyrate did not affect its phosphorylation levels. Considering that dephosphorylation of cdc2 leads to entry to the M phase, the persistent tyrosine phosphorylation of cdc2 may be associated with the S phase accumulation induced by IFN-α and sodium butyrate. In addition, another human CML leukaemic cell line, MEG-01, also showed the S phase accumulation after stimulation with IFN-α plus sodium butyrate. Taken together, our studies reveal a novel effect of sodium butyrate on the S phase accumulation and suggest its clinical application for a combination therapy with IFN-α, leading to a great improvement of clinical effects of IFN-α against CML cells.

**Keywords:** butyrate; IFN-α; S phase accumulation; cdc2; tyrosine phosphorylation

The interferons (IFNs) are cytokines that produce divergent biological activities, including inhibition of viral replication and suppression of tumour growth (Lengyel, 1982). There are two types of IFNs that bind specific receptors (R) and initiate their biological activities. Type I IFNs (α and β) share the same receptor, whereas type II IFN (IFN-γ) uses its own receptor (Farrar and Schreiber, 1993). IFN-α induces distinct sets of genes, which possess interferon-stimulated response element (ISRE)-like DNA sequences (Dale et al, 1989). Ligand-bound type I-receptors associate with Jak1 and tyk2 protein tyrosine kinases (PTKs), which subsequently phosphorylate signal transducer and activator of transcription (Stat) proteins Stat1 and Stat2 (Leung et al, 1995; Stahl et al, 1995; Yan et al, 1996). Phosphorylated Stats migrate to the nucleus, bind to ISRE-like elements and stimulate transcription immediately (Darnell et al, 1994).

Although IFNs have been used for anti-cancer therapy, the mechanisms of their anti-proliferative effects remain to be further defined. Apparently, IFN-α inhibits cell cycle progression in various cancer cells (Gutterman, 1994). IFN-α induces $G_0/G_1$ arrest through the activation of the retinoblastoma protein (pRB) by suppression of its phosphorylation and the down-regulation of *c-myc* gene expression (Einat et al, 1985; Resnitzky et al, 1992). In some other cells, IFN-α induces blockage of the cell cycle at $G_2$/M transition or prolongation of the S phase (Wadler and Schwartz, 1990; Oberg, 1992). So far, little is known about the mechanism for these different effects of IFN-α on inhibition of cell cycle progression. It has been reported that loss or inactivation of the normal $G_1$ checkpoint conferred by pRB is associated with the IFN-α-mediated S phase accumulation (Qin et al, 1997). However, most cancer cells have disruption of the $G_1$ checkpoint and express an intact IFN type I receptor, but IFN-α-induced S phase accumulation is not common. Thus, an unidentified factor(s) appears to be involved in biological effects of IFN-α. From the aspect of cancer therapies, identification of the factors may greatly improve its therapeutic effect of IFN-α. We report here that sodium butyrate augmented IFN-α-induced accumulation of the S phase in human chronic myelocytic leukaemia (CML) K562 cells. During accumulation of cells in the S phase, tyrosine phosphorylation of cdc2 protein was sustained. The persistent phosphorylation of cdc2 correlated well with the accumulation of the S phase. In sharp contrast, IFN-γ neither affected tyrosine phosphorylation of cdc2 nor induced S phase accumulation. Considering that dephosphorylation of cdc2 is required for entry into the $G_2$/M phase, its persistent phosphorylation may be associated with accumulation of the S phase. In addition, sodium butyrate similarly augmented the IFN-α-induced accumulation of the S phase in human CML MEG-01

*Received 4 February 1998*
*Revised 7 July 1998*
*Accepted 26 August 1998*

*Correspondence to:* M Adachi cells. Taken together, these results suggest that sodium butyrate may increase the anti-proliferative effect of IFN-α on CML cells.

### MATERIALS AND METHODS

### Cell culture and reagents

Human CML cell lines K562 and MEG-01 were cultured in Roswell Park Memorial Institute (RPMI)-1640 medium (Gibco, Grand Island, NY, USA) with 10% fetal calf serum (FCS). To evaluate cell viability, dead cells were counted by trypan blue staining method. Human IFN-α and IFN-γ were kindly supplied by Sumitomo Chemical Pharmacy and Shionogi Chemical Pharmacy (Osaka, Japan), respectively. Sodium butyrate was purchased from Sigma Chemical Co. (St Louis, MO, USA).

### Cell cycle analysis

Cell cycle distribution was determined by staining DNA with propidium iodide essentially as described (Adachi et al, 1994). Briefly, cells were stained with propidium iodide (Sigma) and passed through the beam of an argon ion laser turned to 585 nm (FAC/Scan; Becton Dickinson). The resulting fluorescent signal was amplified, recorded and analysed by using a NIH image program for a DNA histogram.

### Antibodies and immunoblotting

Western blot analysis was performed essentially as described (Adachi et al, 1997). Briefly, cells were washed with cold phosphate-buffered saline (PBS) and lysed in 500 μl of a buffer containing 100 mM sodium chloride (NaCl), 2 mM EDTA, 10 mM sodium orthovanadate, 1 mM phenylmethylsulphonyl fluoride (PMSF), 1% NP-40 and 50 mM Tris (pH 7.2). Their protein concentrations were analysed by the Protein Assay kit (BioRad) and each lysate (1 mg/sample) was subjected to immunoprecipitation with anti-cdc2 monoclonal antibody (Transduction Laboratories). The immunoprecipitates were then subjected to sodium dodecyl sulphate polyacylamide gel electrophoresis (SDS-PAGE), followed by electrophoretical transfer to Immobilon (Millipore). The blots were incubated with blocking buffer containing 3% bovine serum albumin (BSA), 10 mM Tris (pH 8.2), 140 mM NaCl and 0.01% $NaN_3$. Blots were then incubated with 1 μg $ml^{-1}$ of anti-cdc2 antibody for 2 h in washing solution (150 mM NaCl, 10 mM Tris (pH 7.5) and 0.01% Tween 20) with 3% FCS, and developed by a standard enhanced chemiluminescence (ECL) method (Amersham, Arlington Heights, IL, USA). After stripping in stripping buffer containing 100 mM 2-mercaptoethanol, 2% SDS and 62.5 mM Tris-HCl pH 6.7 for 0.5 h, the same blot was subsequently incubated with anti-phosphotyrosine 4G10 antibody (UBI).

### RESULTS

### Effects of butyrate and IFN-α on K562 cells

A human CML cell line K562 is capable to undergo erythrocytic differentiation by the stimulation with sodium butyrate (Anderson et al, 1979). Treatment with 1 mM sodium butyrate increased expression of glycophorin-A, a marker of erythrocytes, on K562 cells by 31% (Figure 1A). During differentiation of K562 cells with sodium butyrate, their cell numbers gradually decreased compared with control cells (Figure 1B). When K562 cells were incubated with sodium butyrate for 5 days, their cell number was reduced to 52.8% of that of control cells.

Next, we investigated effects of IFN-α on K562 cells. IFN-α is known to suppress proliferation of various cancer cells, including CML cells (Lengyel, 1982; Ozer et al, 1993). Cell growth curves

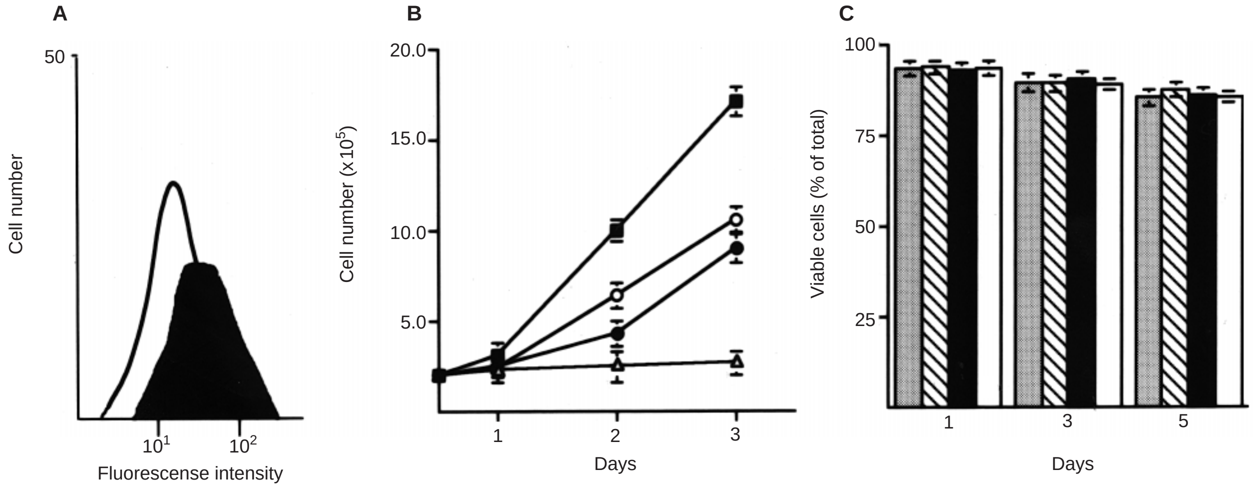

**Figure 1** Effect of IFN-α and sodium butyrate on cell proliferation and viability of K562 cells. (**A**) Expression of glycophorin, a marker for erythroid differentiation, on K562 cells with (shaded) or without (open) 1 mM sodium butyrate. The results were obtained by a two-step reaction with an anti-glycophorin monoclonal antibody (mAb) and a goat-anti-mouse phycoerythrin-labelled antibody. (**B**) K562 cells were treated with 600 IU $ml^{-1}$ of recombinant human IFN-α (❍) or 1 mM sodium butyrate (●) for the indicated number of days. Treatment of K562 cells with IFN-α plus sodium butyrate greatly reduced cell proliferation (△). The growth curve of control K562 cells, which were cultured in 10% FCS, is also shown (■). The mean number of cells from three independent experiments was plotted against the number of days. Standard error values are indicated. (**C**) No significant effect of IFN-α and sodium butyrate on cell viability of K562 cells. K562 cells were treated with IFN-α alone (□), sodium butyrate alone (▩) or IFN-α plus sodium butyrate (■) for the indicated number of days and their cell viability was assessed by trypan blue exclusion assay. Cell viability of control K562 cells is also shown (▧). The mean numbers of the cells were obtained from three independent experiments

*© Cancer Research Campaign 1999*

of K562 cells cultured in medium containing IFN-α with or without sodium butyrate are shown in Figure 1B. The treatment with IFN-α alone slightly suppressed proliferation of K562 cells, but addition of IFN-α plus sodium butyrate further suppressed it, and their increase of cell number was mostly abolished (Figure 1B).

We investigated whether these reagents induced apoptotic cell death of K562 cells. Trypan blue exclusion assay showed that the treatments with IFN-α and/or sodium butyrate did not increase loss of cell viability significantly (Figure 1C). The treatment of K562 cells with IFN-α plus sodium butyrate for 5 days resulted in growth arrest; however, the treatment led to neither distinct morphological changes characteristic of apoptosis (data not shown) nor loss of cell viability.

### Butyrate augments S phase accumulation by IFN-α

As shown above, the treatment with sodium butyrate plus IFN-α inhibited proliferation of K562 cells, but had little effect on their viability. This led us to consider whether the treatment might affect cell cycle progression. Following treatments with either sodium butyrate or IFN-α for 3 days, K562 cells were harvested and subjected to cell cycle analysis. When K562 cells were cultured with IFN-α, a slight but significant increase in the S phase cell population was observed (S phase was 39.6 ± 2.0%) as shown in Figures 2 and 4. Although sodium butyrate alone showed no significant effect on the cell cycle in K562 cells (S phase was 24.0 ± 0.8%), butyrate plus IFN-α treatment markedly increased the S phase cell population (S phase was 52.5 ± 3.7%) compared with the cell population treated with IFN-α alone. The S phase increase was accompanied by a decrease in the percentage of cells in the G1 phase (65% to 27%).

We next investigated the time-course of S phase accumulation following the treatment with sodium butyrate plus IFN-α. As shown in Figure 3, no obvious change was seen after 1 day of the treatment, but the S phase population began to accumulate at 3 days, and a significant increase of S phase accumulation was seen (214% of control) with a corresponding decrease in the G1 phase (41.7% of control). By 5 days after the treatment, the percentage of cells in the S phase was slightly decreased but still higher than that of control cells. Similar results were obtained in three separate experiments.

### IFN-γ had no effect on cell cycle in K562 cells

We also investigated whether IFN-γ induces accumulation of the S phase or affects the action of sodium butyrate in K562 cells. Following treatment of K562 cells with IFN-γ alone or IFN-γ plus sodium butyrate for 3 days, their cell cycle populations were monitored. The IFN-γ stimulations either with or without sodium butyrate showed no significant changes in the percentages of cells in the S phase and other cell cycle phases. When K562 cells were cultured with IFN-γ plus sodium butyrate, the percentage of cells in the S phase was 23.1%, while the percentage of K562 cells treated with IFN-γ alone was 24.8% (Figure 4A). Thus, accumulation of the S phase was specifically induced by IFN-α and augmentation of S phase accumulation by sodium butyrate depended upon IFN-α-mediated signalling.

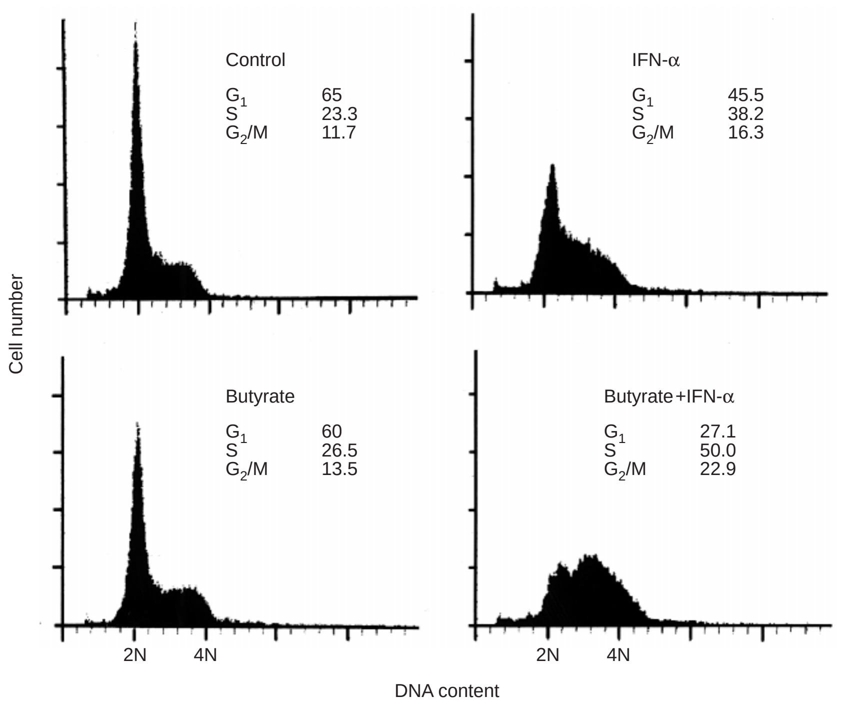

**Figure 2** DNA histograms showing the cell cycle distribution of K562 cells. K562 cells untreated (control) or treated with IFN-α and/or sodium butyrate for 3 days were stained with propidium iodide and subjected to FACS analysis. Each of the cell cycle profiles contains the percentage of cells in different stages of the cycle, which was evaluated with the NIH image analysis program

 *© Cancer Research Campaign 1999*

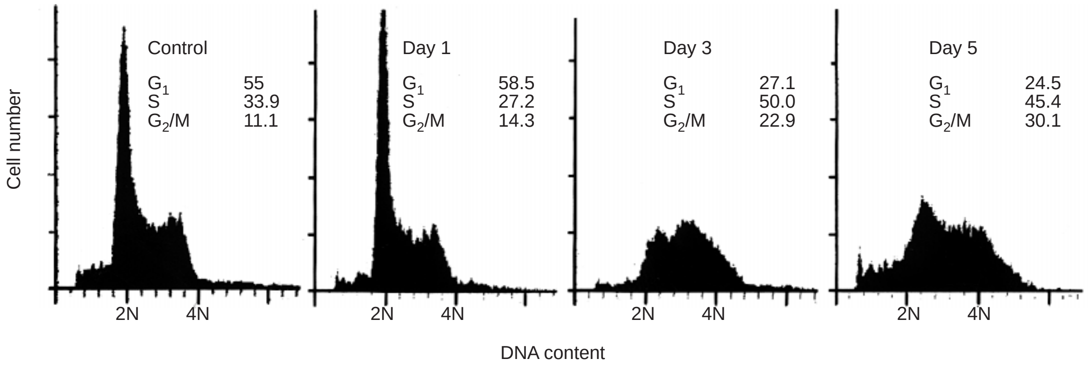

**Figure 3** Time course of the S phase accumulation of K562 cells treated with IFN-α plus sodium butyrate. DNA histograms showing the cell cycle distribution of K562 cells treated with both 600 IU ml$^{-1}$ of IFN-α and 1 mM sodium butyrate for the indicated number of days. Each sample was stained with propidium iodide and subjected to FACS analysis. Each of the cell cycle profiles contains the percentage of cells in different stages of the cycle, which was evaluated with NIH image analysis

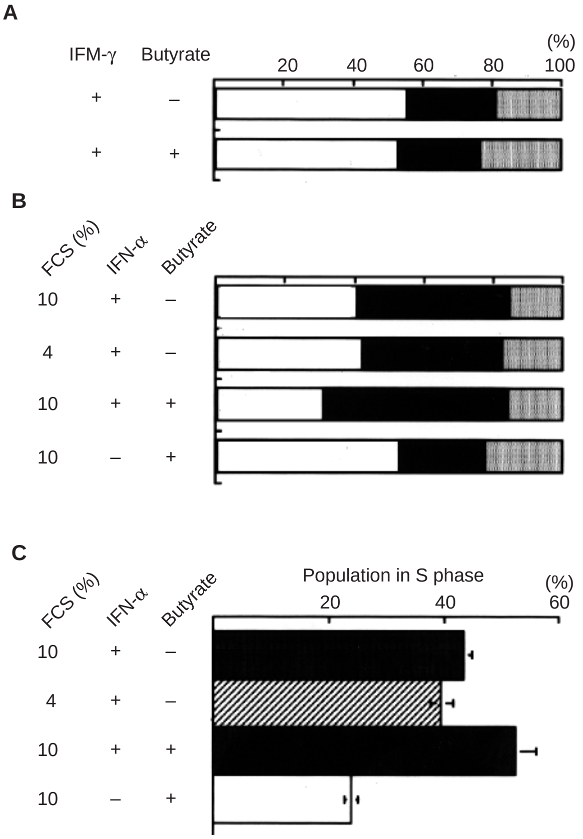

**Figure 4** Cell cycle distribution of K562 cells after treatment with IFN-α, IFN-γ and sodium butyrate. (**A**) The percentages of cells in different stages of the cycle according to the cell cycle profiles of K562 cells treated with IFN-γ with or without sodium butyrate for 3 days. (**B**) Cell cycle distribution of K562 cells grown under various conditions for 3 days. K562 cells were cultured in medium containing 4% FCS plus IFN-α, or treated with IFN-α and/or sodium butyrate. Each bar contains $G_0/G_1$ (open column), S (closed column) and $G_2/M$ (shaded column) phase populations. (**C**) The percentages of K562 cells in the S phase under various conditions described in (**B**). The mean numbers of their percentages were obtained from three independent experiments. Standard error values are indicated

### Low serum concentration did not augment S phase accumulation induced by IFN-α

As shown in Figure 1, the sodium butyrate treatment slightly suppressed cell proliferation of K562 cells. The suppression might affect the S phase accumulation by IFN-α. To exclude this possibility, we investigated the effect on S phase accumulation induced by IFN-α of culture in a medium containing 4% FCS. Culture of K562 cells in 4% FCS-containing medium showed a growth curve similar to that seen in their culture with 1 mM sodium butyrate. After 3-day culture of K562 cells ($2.0 \times 10^5$ cells ml$^{-1}$), the cell density of K562 cells in 4% FCS-containing medium was $4.1 \pm 0.22 \times 10^5$ cells ml$^{-1}$, while the density in sodium butyrate-containing medium was $4.3 \pm 0.35 \times 10^5$ cells ml$^{-1}$. When K562 cells were cultured with 4% FCS plus IFN-α, their cell cycle populations (S phase was 43.3 ± 1.8%) exhibited a pattern similar to that seen in cells treated with IFN-α alone (S phase was 39.6 ± 2.0%) as shown in Figure 4. Thus, augmentation of S phase accumulation by sodium butyrate seemed not to be associated with suppression of cell proliferation, but rather the augmentation depended upon actions of sodium butyrate in a specific manner.

### Persistent tyrosine phosphorylation of cdc2 by IFN-α and butyrate, but not by IFN-γ and butyrate

During progression of the cell cycle, 34 kDa protein kinase cdc2 and cyclin B play key roles in regulation of the cell cycle (Nurse, 1990). The level of cdc2 is constant throughout the cell cycle, but its kinase activity is sharply periodic, rising to a peak at the $G_2/M$ transition (Moreno et al, 1989). Activation of cdc2 kinase is required for checkpoints preventing entry into mitosis before completion of the S phase (Enoch and Nurse, 1990). The activation is determined by dephosphorylation of a tyrosine (Y15) within its catalytic site (Gould and Nurse, 1989), which is brought about by phosphatase cdc25 (Straufeld et al, 1991). To investigate whether S phase accumulation induced by IFN-α plus sodium butyrate is associated with regulation of cdc2 activity, tyrosine phosphorylation levels of cdc2 were examined by Western blotting using a monoclonal antibody that specifically binds to tyrosine phosphorylated cdc2. When K562 cells were cultured with or without IFN-α and/or sodium butyrate for 3 days, the treatment with IFN-α increased the tyrosine phosphorylation level of cdc2, and the level was the highest in K562 cells with IFN-α plus

© Cancer Research Campaign 1999

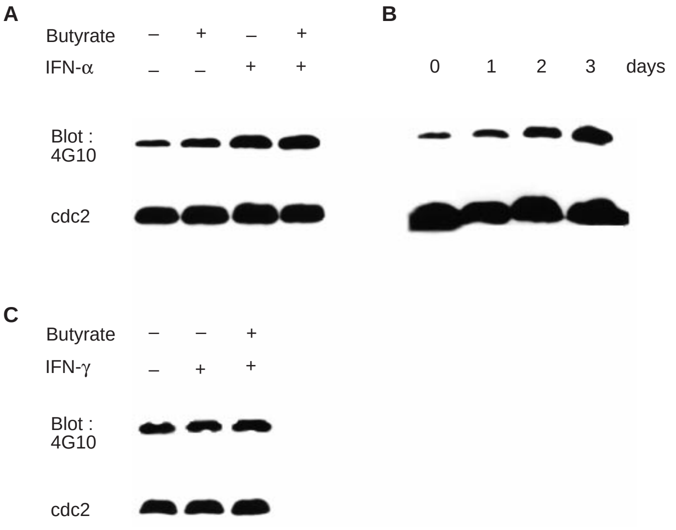

**Figure 5** Tyrosine phosphorylation of cdc2. (**A**) IFN-α and sodium butyrate treatment sustained tyrosine phosphorylation of cdc2 in K562 cells. K562 cells were cultured with or without IFN-α and/or sodium butyrate for 3 days, and tyrosine phosphorylation levels of their cdc2 were analysed. (**B**) Time-course of tyrosine phosphorylation of cdc2. K562 cells were cultured with or without IFN-α and sodium butyrate for the indicated number of days. (**C**) IFN-γ and sodium butyrate treatment did not affect tyrosine phosphorylation of cdc2 in K562 cells. K562 cells were cultured with or without IFN-γ alone or with sodium butyrate for 3 days, and tyrosine phosphorylation levels of their cdc2 were analysed. Total cell lysates were immunoprecipitated with an anti-cdc2 antibody and tyrosine phosphorylation levels of the immunoprecipitates were analysed by immunoblotting using an anti-phosphotyrosine antibody 4G10 at 1 μg ml$^{-1}$

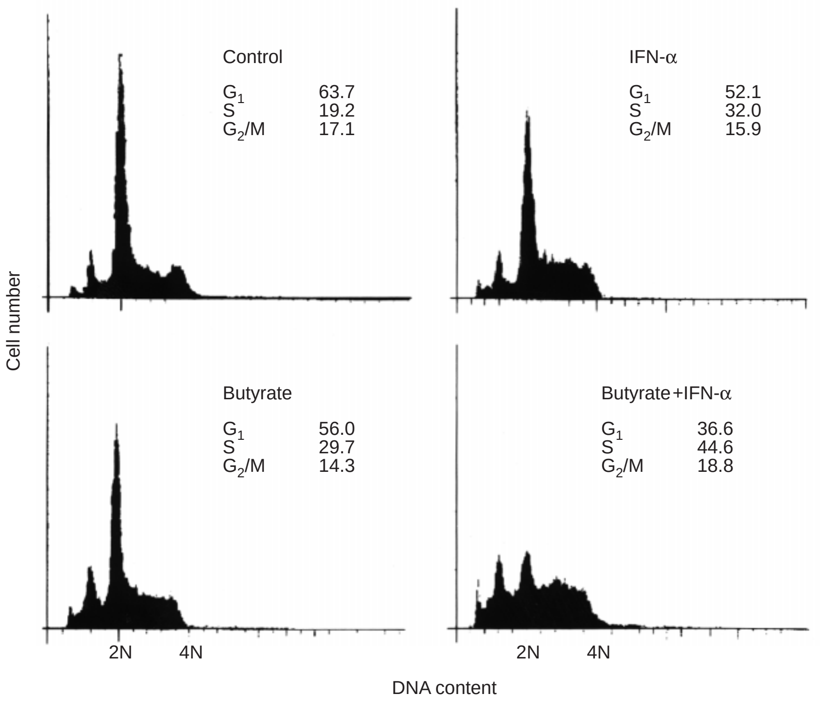

**Figure 6** DNA histograms showing the cell cycle distribution of MEG-01 cells. MEG-01 cells untreated (control) or treated with IFN-α and/or sodium butyrate for 3 days were stained with propidium iodide and subjected to FACS analysis. Each of the cell cycle profiles contains the percentage of cells in different stages of the cycle, which was evaluated with the NIH image analysis program sodium butyrate (Figure 5A). When the levels were normalized for their protein amount, the levels of K562 cells treated with IFN-α and IFN-α plus sodium butyrate were increased by 2.0-fold and 2.6-fold compared with control cells, respectively. The time-course of tyrosine phosphorylation levels of cdc2 revealed that the levels gradually increased by 3 days (Figure 5B). In contrast, the treatment with IFN-γ plus sodium butyrate did not affect the tyrosine phosphorylation levels of cdc2 significantly (Figure 5C).

### Butyrate also augmented IFN-α-induced S phase accumulation in MEG-01

To explore whether sodium butyrate augments the IFN-α-induced S phase accumulation in other CML cells, we investigated the effect of sodium butyrate in MEG-01 cells, which have been established from a patient with Philadelphia chromosome-positive CML in blast crisis (Ogura et al, 1985). When MEG-01 cells were

© Cancer Research Campaign 1999

cultured with IFN-α, a slight but significant increase in the S phase cell population was observed (S phase was 32.0%) as shown in Figure 6. Sodium butyrate also showed a slight effect on the cell cycle in MEG-01 cells (S phase was 29.7%), but the butyrate plus IFN-α treatment markedly increased the S phase cell population (S phase was 44.6%) compared with the cell population treated with IFN-α alone. The S phase increase was accompanied by a decrease in the percentage of cells in the $G_1$ phase (63.7% to 36.6%). Thus, the butyrate plus IFN-α treatment can increase the S phase cell population of at least two CML cell lines.

### DISCUSSION

In this report, we have shown that sodium butyrate augments IFN-α-induced S phase accumulation. Although sodium butyrate reduced cell proliferation of K562 cells, this effect seemed to be marginal with respect to its enhancement of IFN-α-induced S phase accumulation, since reduced cell proliferation due to incubation of K562 cells in medium containing a low FCS concentration had no significant effect on IFN-α-induced S phase accumulation. Although sodium butyrate can induce apoptosis in many cancer cells (Calabresse et al, 1993; Hague et al, 1993; McBain et al, 1996), this reagent neither induced nor augmented apoptosis when co-incubated with IFN-α in K562 cells, at least during incubation for 5 days. It is not clear how sodium butyrate augments IFN-α-induced S phase accumulation. A previous report showed a positive interaction between IFN-α and chemotherapy (Balkwill et al, 1984). Cyclophosphamide, an alkylating agent, caused accumulation in the $G_2$ and S phases. The addition of IFN-α with cyclophosphamide delayed the $G_2$ block and markedly increased the cell population in the S phase. Considering the effect of alkylating agents as inhibitors of DNA synthesis, it is conceivable that combination of cyclophosphamide with IFN-α increased S phase accumulation. In contrast, there has been no report suggesting that sodium butyrate is capable of inhibiting DNA synthesis. Sodium butyrate is a short chain fatty acid produced by bacterial degradation of poorly fermentable dietary fibres in the colorectum (Reeder et al, 1993), and is known to inhibit splicing of the *c-myc* gene (Krupitza et al, 1995) and several signal transduction pathways, i.e. inhibition of the release of $Ca^{2+}$ from intracellular stores and casein kinase II activity (Russo et al, 1997). Since the treatment of K562 cells with sodium butyrate alone reduced cell proliferation, the reagent may inhibit growth signals mediated by FCS. However, exposure of K562 cells to sodium butyrate alone did not affect the cell cycle significantly and its effect on S phase accumulation totally depended upon IFN-α-mediated signals. As IFN-α and sodium butyrate synergistically suppressed the proliferation of K562 cells, they may cooperate with the function for suppression of cell cycle progression and cause accumulation of the population in the S phase.

Sodium butyrate is an erythroid differentiation inducer of K562 cells. Thus, its augmentation of the S phase may be associated with differentiation of K562 cells. To investigate this possibility, we examined the cell populations of the cell cycle following treatment with TPA, a megakaryocytic differentiation inducer for K562 cells. TPA and/or IFN-α treatment had no significant effect on the S phase population (data not shown). In HL60 cells, TPA and RA, differentiation inducers of monocytes and myeloid cells, respectively, could not augment S phase accumulation (data not shown). Thus, differentiation events were not directly involved in the augmentation of S phase accumulation, but rather a specific action of the reagent might have been involved in the augmentation. Thus, sodium butyrate is a unique differentiation inducer which can augment S phase accumulation.

Early studies demonstrated that IFN-α induces $G_0/G_1$ arrest in Daudi Burkitt's lymphoma or M1 myeloid cells (Einat et al, 1985; Tiefenbrun et al, 1996). However, IFN-α also induces S phase accumulation in a variety of cancer cells (Qin et al, 1997). Though molecular mechanisms for these different effects of IFN-α remain to be clarified, the $G_1$ checkpoint function is thought to be a major factor to determine different effects of IFN-α; $G_0/G_1$ arrest occurs in cancer cells containing functional pRB, a key molecule for the $G_1$ checkpoint, whereas S phase accumulation occurs in cancer cells lacking functional pRB. K562 cells are known to express functional pRB, which is required for erythroid differentiation (Bergh et al, 1997). This implies that IFN-α can induce S phase accumulation even in cells expressing functional pRB. Considering that K562 cells lack p53 expression (Sugimoto et al, 1992), the loss of p53 function is likely to induce uncontrolled CDK activity, leading to aberrant phosphorylation of pRB, which may explain why IFN-α induces S phase accumulation in K562 cells.

cdc2 plays key roles in regulation of the cell cycle and its activity is predominantly regulated by tyrosine phosphorylation levels (Gould and Nurse, 1989; Nurse, 1990). In the late $G_1$ phase, cdc2 is tyrosine phosphorylated by wee1 or mik1 (Featherstone and Russell, 1991; Lundgren et al, 1991) and the phosphorylated cdc2 is then dephosphorylated by cdc25 before entry into the M phase (Straufeld et al, 1991). Thus, dephosphorylation of cdc2 seems to be required for $G_2$/M transition, though tyrosine phosphorylation of cdc2 has not been demonstrated to cause S phase arrest in mammalian cells (Sherr, 1993). To explore the mechanisms of IFN-α-induced S phase accumulation and its augmentation by sodium butyrate in K562 cells, we investigated tyrosine phosphorylation levels of cdc2. The IFN-α treatment inhibited dephosphorylation of cdc2, and IFN-α plus sodium butyrate further inhibited the dephosphorylation, resulting in persistent phosphorylation of cdc2. Considering the tight correlation between S phase accumulation and elevated phosphorylation levels of cdc2, it is conceivable that tyrosine phosphorylation of cdc2 may be a target of IFN-α. This idea is supported by a recent report that IFN-α can suppress cdc25 expression (Tiefenbrun et al, 1996), which is responsible for dephosphorylation of cdc2. Alternatively, a target of IFN-α and sodium butyrate would be the replication machinery and subsequently result in persistent tyrosine phosphorylation of cdc2, since cdc2 is known to be phosphorylated in response to DNA damage and may function to block inappropriate entry into mitosis (Jin et al, 1996). In addition, the IFN-α plus sodium butyrate treatment may inhibit the cell cycle more generally, since the S phase accumulation (S phase cell population was approximately 50%) appears not to fully explain the complete loss of cell proliferation (Figure 1B), and cell population at the $G_2$/M phase also increased in response to the treatment. Obviously, it is important to clarify the precise molecular machinery of IFN-α-induced S phase accumulation and its augmentation by sodium butyrate.

Our data clearly showed that sodium butyrate can enhance the effect of IFN-α by augmentation of S phase accumulation. Though sodium butyrate has very high toxicity in vivo, the effectiveness of sodium butyrate derivatives for colon cancer patients is now under

© Cancer Research Campaign 1999

investigation in extensive clinical trials. If a non-toxic derivative of sodium butyrate is available, its combination with IFN-α may greatly improve the anti-tumour effect of IFN-α.

### ACKNOWLEDGEMENTS

We thank Mr Kim Barrymore for editorial work. This work was supported in part by a Grant-in-Aid for the Second Term Comprehensive 10-Year Strategy for Cancer Control and Cancer Research and a Grant-in-Aid for Scientific Research on Priority Areas (Cancer) from the Ministry of Education, Science and Culture, Japan.

### REFERENCES

Adachi M, Sekiya M, Ishino M, Sasaki H, Hinoda Y, Imai K and Yachi A (1994) Induction of protein tyrosine phosphatase LC-PTP by IL-2 in human T cells: LC-PTP is an early response gene. *FEBS Let* **338**: 47–52

Adachi M, Ishino M, Torigoe T, Minami Y, Matozaki T, Miyazaki T, Taniguchi T, Hinoda Y and Imai K (1997) Interleukin-2 induces tyrosine phosphorylation of SHP-2 through IL-2 receptor β chain. *Oncogene* **14**: 1629–1633

Anderson LC, Jokinen M and Gahmberg CG (1979) Induction of erythroid differentiation in the human leukemia cell line K562. *Nature (Lond)* **278**: 364–365

Balkwill FR, Mowshowitz S, Seilman SS, Moodie EM, Griffin DB, Fantes KH and Wolf CR (1984) Positive interactions between interferon and chemotherapy due to direct tumor action rather than effects on host drug-metabolizing enzymes. *Cancer Res* **44**: 5249–5255

Bergh G, Ehinger M, Olofsson T, Baldetorp B, Johnsson E, Brycke H, Lindgren G, Olsson I and Gullberg U (1997) Altered expression of the retinoblastoma tumor-suppressor gene in leukemic cell lines inhibits induction of differentiation but not $G_1$-accumulation. *Blood* **89**: 2938–2950

Calabresse C, Venturini L, Ronco G, Villa P, Chomienne C and Belpomme D (1993) Butyric acid and its monosaccharide ester induce apoptosis in the HL-60 cell line. *Biochem Biophys Res Commun* **195**: 31–38

Dale TC, Iman AMA, Kerr IM and Stark GR (1989) Rapid activation by interferon alpha of a latent DNA binding protein present in the cytoplasm of untreated cells. *Proc Natl Acad Sci USA* **86**: 1203–1207

Darnell JE, Kerr IM and Stark GR (1994) Jak-STAT pathways and transcriptional activation in response to IFNs and other extracellular signaling proteins. *Science* **264**: 1415–1421

Einat M, Resnitzky D and Kimchi A (1985) Close link between reduction of c-*myc* expression by interferon and $G_0/G_1$ arrest. *Nature* **313**: 597–600

Enoch T and Nurse P (1990) Mutation of fission yeast cell cycle control genes abolishes dependence of mitosis on DNA replication. *Cell* **60**: 665–673

Farrar MA and Schreiber RD (1993) The molecular cell biology of interferon-gamma and its receptor. *Annu Rev Immunol* **11**: 571–579

Featherstone C and Russell P (1991) Fission yeast p107$^{wee1}$ mitotic inhibitor is a tyrosine/serine kinase. *Nature* **349**: 808–811

Gould KL and Nurse P (1989) Tyrosine phosphorylation of the fission yeast cdc2$^{+}$ protein kinase regulates entry into mitosis. *Nature* **342**: 39–45

Gutterman JU (1994) Cytokine therapeutics: lessons from interferon α. *Proc Natl Acad Sci USA* **91**: 1198–1205

Hague A, Manning AM, Hanlon KA, Nuschtscha LI, Hart D and Paraskeva C (1993) Sodium butyrate induces apoptosis in human colonic tumor cell lines in a p53-independent pathway: implications for the possible role of dietary fibre in the prevention of large-bowel cancer. *Int J Cancer* **55**: 498–505

Jin P, Gu Y and Morgan DO (1996) Role of inhibitory CDC2 phosphorylation in radiation-induced G2 arrest in human cells. *J Cell Biol* **134**: 963–970

Krupitza G, Harant H, Dittrich E, Szekeres T, Huber H and Dittrich C (1995) Sodium butyrate inhibits c-myc splicing and interferes with signal transduction in ovarian carcinoma cells. *Carcinogenesis* **16**: 1199–1205

Lengyel P (1982) Biochemistry of interferons and their actions. *Annu Rev Biochem* **51**: 251–282

Leung S, Qureshi SA, Kerr IM, Darnell JE and Stark GR (1995) Role of STAT2 in the alpha interferon signalling pathway. *Mol Cell Biol* **15**: 1312–1317

Lundgren K, Walworth N, Booher R, Dembski M, Kirschner M and Beach D (1991) mik1 and wee1 cooperate in the inhibitory tyrosine phosphorylation of cdc2. *Cell* **64**: 1111–1122

McBain JA, Eastman A, Simmons DL, Pettit GR and Mueller GC (1996) Phorbol ester augments butyrate-induced apoptosis of colon cancer cells. *Int J Cancer* **67**: 715–723

Moreno S, Hayles J and Nurse P (1989) Regulation of p34$^{cdc2}$ protein kinase during mitosis. *Cell* **58**: 361–372

Nurse P (1990) Universal control mechanism regulating onset of M phase. *Nature* **344**: 503–508

Oberg K (1992) The action of interferon α on human carcinoid tumors. *Semin Cancer Biol* **3**: 35–41

Ogura M, Morishima Y, Ohno R, Kato Y, Hirabayashi N, Nagura H and Saito H (1985) Establishment of a novel human megakaryoblastic leukemia cell line, MEG-01, with positive Philadelphia chromosome. *Blood* **66**: 1384–1392

Ozer H, George SL, Schiffer CA, Rao K, Rao PN, Wurster-Hill DH, Arther DD, Powell B, Gottlieb A, Peterson BA, Rai K, Testa JR, LeBeau M, Tantravahi R and Bloomfield CD (1993) Prolonged subcutaneous administration of recombinant α-2β interferon in patients with previously untreated Philadelphia chromosome-positive chronic phase myelogenous leukemia: effect on remission duration and survival. Cancer and Leukemia Group B Study 8583. *Blood* **82**: 2975–2984

Qin X-Q, Runkel L, Deck C, DeDios C and Barsoum J (1997) Interferon-β induces S phase accumulation selectively in human transformed cells. *J Interferon Cytokine Res* **17**: 355–367

Reeder JA Dickinson JL, Chenevix-Trench G and Antalis TM (1993) Sodium butyrate differentially modulates plasminogen activator inhibitor type-I, urokinase plasminogen activator and its receptor in a human colon carcinoma cell. *Teratog Carcinog Mutagen* **13**: 75–88

Resnitzky D, Tiefenbrun N, Berissi H and Kimchi A (1992) Interferon and interleukin 6 suppress phosphorylation of the retinoblastoma protein in growth-sensitive hematopoietic cells. *Proc Natl Acad Sci USA* **89**: 402–406

Russo GL, Pietra VD, Mercurio C, Ragione FD, Marshak DR, Oliva A and Zappia V (1997) Down-regulation of protein kinase CKII activity by sodium butyrate. *Biochem Biophys Res Commun* **233**: 673–677

Sherr CJ (1993) Mammalian G1 cyclins. *Cell* **73** 1059–1065

Stahl N, Farruggella TJ, Boulton TG, Zhong Z, Darnell JE and Yancopoulos GD (1995) Choices of STATs and other substrates specified by modular tyrosine-based motifs in cytokine receptor. *Science* **267**: 1349–1353

Strausfeld U, Labbé JC, Fesquet D, Cavadore JC, Picard A, Sadhu K, Russell P and Dorée M (1991) Dephosphorylation and activation of a p34$^{cdc2}$/cyclin B complex in vitro by human CDC25 protein. *Nature* **351**: 242–245

Sugimoto K, Toyoshima H, Sakai R, Miyagawa K, Hagiwara K, Ishikawa F, Takaku F, Yazaki Y and Hirai H (1992) Frequent mutations in the p53 gene in human myeloid leukemia cell lines. *Blood* **79**: 2378–2383

Tiefenbrun N, Melamed D, Levy N, Resnitzky D, Hoffmann I, Reed S and Kimchi A (1996) Alpha interferon suppresses the cyclin D3 and cdc25A genes, leading to a reversible G0-like arrest. *Mol Cell Biol* **16**: 3934–3944

Wadler S and Schwartz EL (1990) Antineoplastic activity of the combination of interferon and cytotoxic agents against experimental and human malignancies: a review. *Cancer Res* **50**: 3473–3486

Yan H, Krishnan K, Greenlund AC, Gupta S, Lim JTE, Schreiber RD, Schindler CW and Krolewski JJ (1996) Phosphorylated interferon-a receptor I subunit (IFNAR1) acts as a docking site for the latent form of the 113 kDa Stat2 protein. *EMBO J* **15**: 1064–1074

 *© Cancer Research Campaign 1999*
